# Bacterial but Not Fungal Gut Microbiota Alterations Are Associated With Common Variable Immunodeficiency (CVID) Phenotype

**DOI:** 10.3389/fimmu.2019.01914

**Published:** 2019-08-13

**Authors:** Kristýna Fiedorová, Matěj Radvanský, Juraj Bosák, Hana Grombiříková, Eva Němcová, Pavlína Králíčková, Michaela Černochová, Iva Kotásková, Matej Lexa, Jiří Litzman, David Šmajs, Tomáš Freiberger

**Affiliations:** ^1^Centre for Cardiovascular Surgery and Transplantation, Brno, Czechia; ^2^Central European Institute of Technology, Masaryk University, Brno, Czechia; ^3^Department of Clinical Immunology and Allergology, Faculty of Medicine, Masaryk University, Brno, Czechia; ^4^Faculty of Informatics, Masaryk University, Brno, Czechia; ^5^Department of Biology, Faculty of Medicine, Masaryk University, Brno, Czechia; ^6^Department of Allergology and Clinical Immunology, Faculty of Medicine, Charles University and University Hospital in Hradec Kralove, Hradec Kralove, Czechia; ^7^Department of Clinical Immunology and Allergology, St. Anne's University Hospital in Brno, Brno, Czechia

**Keywords:** CVID, IgA, gut microbiota, gut microbiome, gut mycobiota, gut mycobiome, fungal microbiota, fungal microbiome

## Abstract

Common Variable Immunodeficiency (CVID) is the most frequent symptomatic immune disorder characterized by reduced serum immunoglobulins. Patients often suffer from infectious and serious non-infectious complications which impact their life tremendously. The monogenic cause has been revealed in a minority of patients so far, indicating the role of multiple genes and environmental factors in CVID etiology. Using 16S and ITS rRNA amplicon sequencing, we analyzed the bacterial and fungal gut microbiota, respectively, in a group of 55 participants constituting of CVID patients and matched healthy controls including 16 case-control pairs living in the same household, to explore possible associations between gut microbiota composition and disease phenotype. We revealed less diverse and significantly altered bacterial but not fungal gut microbiota in CVID patients, which additionally appeared to be associated with a more severe disease phenotype. The factor of sharing the same household impacted both bacterial and fungal microbiome data significantly, although not as strongly as CVID diagnosis in bacterial assessment. Overall, our results suggest that gut bacterial microbiota is altered in CVID patients and may be one of the missing environmental drivers contributing to some of the symptoms and disease severity. Paired samples serving as controls will provide a better resolution between disease-related dysbiosis and other environmental confounders in future studies.

## Introduction

Common Variable Immunodeficiency (CVID) is the most frequent symptomatic immune disorder, estimated to affect 1 in 25,000 people worldwide, although the prevalence can vary across different countries (1, 2). CVID includes clinically and genetically heterogeneous disorders characterized by reduced serum immunoglobulins IgG, IgA, and inconstantly also IgM. As a result of a defect in antibody production, most patients suffer from severe, recurrent infections, mainly of the respiratory and gastrointestinal tract, and have impaired vaccine responses (3, 4). These often manifest as autoimmune complications or inflammatory conditions. Malignancy occurs more frequently in CVID than in the general population (5). Owing to clinical heterogeneity, the disease cannot be determined by a single clinical or laboratory feature. Various diagnostic criteria have been proposed for CVID diagnosis (1, 3, 6, 7). The monogenic cause was revealed in <10% of patients so far, indicating both genetic and environmental factors' contribution in CVID etiology (3, 8).

Recently, human gut microbiota research and its implications in health and disease has attracted tremendous attention. Technological progress in high throughput sequencing has enabled us to associate alterations in gut microbiota composition to a wide variety of human diseases (9, 10) and the expanding knowledge of how gut microbiota affects the host led to new clinical procedure development (11). Recent murine and human studies found gut microbiota to be a crucial factor in shaping and modulating immune system responses (12–14). Gut microbiota modulates the host's immune system via its structural components and metabolites (15, 16). Microbiota-derived metabolites maintain a homeostatic environment of mucus and trigger different immune gene transcription (17, 18). The immune system preserves gut homeostasis and regulates commensal microbiota via immunoglobulin A (IgA) antibodies (19). Schofield and Palm suggested that IgA shapes the gut microbiota in a similar way to how it protects against pathogens in the context of specific species growth restriction (20). Kubinak and Round observed that IgA preferentially targets, and thus limits the levels of microorganisms found in the mucosa while promoting overall microbial diversity via antibody-mediated immunoselection (AMIS), nevertheless the exact mechanisms are poorly understood (21).

Impairing IgA antibody production has been associated with reduced microbiota diversity, and imbalanced microbiota composition resulted in systemic immune activations in mice models (22, 23). One of the possible explanations is an increased opportunity for microbial translocation due to a lack of IgA, which leads to local mucosal inflammation (24). Thus, it has been hypothesized that gut microbiota might be one of the environmental drivers in CVID pathophysiology. To date, only few studies have attempted to describe gut microbiota associations with CVID or selective IgA deficiency syndrome (25–28). Jørgensen et al. showed a positive correlation between disease severity expressed by complication occurrence with higher microbiota dysbiosis and elevated immune activation markers alongside increased lipopolysaccharide (LPS) levels (26). Shulzhenko et al. observed lower mucosal IgA levels in CVID patients suffering from enteropathy than CVID patients without enteropathy and identified three different bacterial taxa that potentially contribute to CVID enteropathy (28).

These studies' results have provided a valuable insight into immunological processes occurring in CVID. However, they have not lead to a clear answer whether gut microbiota composition is causative or a consequence of a CVID phenotype, and thus further studies are needed to elucidate the true effects of low IgA levels. In addition, all studies have been focused on the microbiota's bacterial part and knowledge about fungal microbiota (mycobiota) contribution to CVID etiology is completely lacking. The mycobiota role is relevant in mediating tissue homeostasis (29, 30) and its dysbiosis has been linked to various pathological conditions as well (31, 32). Furthermore, none of the mentioned studies used patients' partners living in the same household as healthy controls to alleviate the impact of environmental cofounders on gut microbiota composition, which is very variable (33).

In this study, we attempted to expand on bacterial gut microbiota knowledge and, for the first time, fungal gut microbiota composition and its association with disease pathogenesis in CVID patients using 16S and ITS rRNA amplicon sequencing. To decrease the impact of the various environmental factors on gut microbiota composition, we also examined case-control couples living in the same household.

## Methods

This study was approved by the Ethic Committee of the Faculty of Medicine, Masaryk University (Protocol no. 37/2016). All enrolled subjects provided written informed consent.

### Subject Recruitment and Sample/Data Collection

CVID patients (*n* = 27) fulfilling International Consensus Document (ICON) diagnostic criteria for CVID (3) were recruited from St. Anne's University Hospital in Brno and the University Hospital Hradec Kralove in the Czech Republic, and characterized according to Ameratunga (1) and Chapel (34) classifications (Supplementary Table S1). Nine patients were treated with regular intravenous immunoglobulin substitution (IVIg) and 18 with subcutaneous immunoglobulin substitution (SCIg). Five patients (18.5%) were treated with immunosuppressive medication. Twenty-two (81.5%) patients suffered from one or more of the following complications: bronchiectasis (*n* = 5), autoimmunity (*n* = 9), splenomegaly (*n* = 18), chronic diarrhea (*n* = 3), atrophic gastritis (*n* = 5), and nodular hyperplasia (*n* = 2); where 16 patients were not examined for the latter two conditions. Healthy controls (*n* = 28) included an age-, sex-, and BMI- matched cohort. Together, 27 CVID patients and 28 healthy controls formed the “ALL” group. Out of the “ALL” group, 16 healthy individuals shared the same household with 16 CVID patients as theirs partners, representing the “PAIRS” subgroup (*n* = 32). The clinical characteristics for the study cohorts are summarized in Table 1.

**Table 1:** General characteristics of study groups (ALL, PAIRS) and cohorts (CVID, CONTROLS): (a) *T*-test; (b) Fisher's exact test.

	**ALL (*n* = 55)**			**PAIRS (*n* = 32)**		
	**CVID (*n* = 27)**	**CONTROLS (*n* = 28)**	***p*-value**	**CVID (*n* = 16)**	**CONTROLS (*n* = 16)**	***p*-value**
Age in years	45.8 ± 12	44.8 ± 12.2	0.7505^a^	45.4 ± 11.9	44.8 ± 10.9	0.8818^a^
Mean ± SD (range)	(26–70)	(23–67)		(26–66)	(25–67)	
Male (%)	37	42.9	0.7848^b^	31.3	68.8	0.0756^b^
BMI	24.8 ± 3.7	26.7 ± 4.6	0.1032^a^	24.7 ± 4.1	26.8 ± 4.1	0.1747^a^
Mean ± SD (range)	(17.7–33.1)	(19.8–38.6)		(18.8–33.1)	(20.1–33.3)	
Smokers; Ex-smokers (*n*)	0;6	1;6	1^b^	0;4	3;1	0.1429^b^
ATB last year (>1 month) (*n*)	14	4	**0.0041**^**b**^	7	1	**0.0372**^**b**^

*P-values < 0.05 (bold) are considered significant*.

All participants provided self-report questionnaire data along with a fecal sample in a sterile container, according to the standardized International Human Microbiome Standards (IHMS) protocol SOP 03 V1 (35) recommended by the International Human Microbiome Consortium. Participants were excluded if they had been treated with antibiotics <1 month prior to sampling. Stool samples were accurately weighed to 200 mg aliquots and frozen at −80°C within 24 h of collection. CVID patients' IgG, IgA, and IgM serum levels were measured during routine medical visits on the day of stool sample collection.

### DNA Extraction and Quantification

Fecal samples were processed using the current standard operating procedure, IHMS protocol Q (36), with minor modifications. Briefly, a frozen aliquot (200 mg) of each sample was thawed and homogenized with 0.6 g of sterile 0.1 and 0.5 mm diameter zirconia beads (BioSpec, Inc., USA) along with 1 mL ASL lysis buffer (Qiagen, Germany). Sample homogenization was undertaken on the Vortex-Genie 2 mixer (MO BIO Laboratories, Inc., USA) for 10 min and the RNase incubation step was omitted. DNA concentration and purity were determined via 260/280 and 260/230 ratios measured on the NanoDrop 1000 (Thermo Fisher Scientific, USA). DNA eluates were stored at −20°C until processing. Sterile water (B. Braun Medical, Inc., Germany) was used as no template control in each DNA extraction round (*n* = 9).

### Library Preparation and Sequencing

The fecal and control samples were profiled by high-throughput amplicon sequencing using the Illumina MiSeq platform (Illumina, USA). The V3-V4 region of the bacterial *16S rRNA* gene was amplified using the primer pair (Bakt_341F/Bakt_805R) containing Illumina adapter sequences (37). Primer pairs ITS1F/ITS2 recommended by the Earth Microbiome Project^1^ with unique barcode sequences designed in our laboratory (38) were used to amplify the fungal internal transcribed spacer region 1 (ITS1) of the rRNA operon. The 16S Library was constructed according to the “16S Metagenomic Sequencing Library Preparation protocol” (37). The ITS1 Library was constructed in a similar manner to the 16S Library, with minor modifications as described previously (38). As a positive control for the sequencing process, the Human Microbiome Project mock community HM-783D (obtained through BEI Resources, NIAID, NIH) also underwent PCR alongside samples and no template controls.

### Bioinformatics

Sequence data analysis for both libraries was processed using Quantitative Insights Into Microbial Ecology (QIIME) pipeline (v.1.9.1.) (39). The ITS1 read pairs were demultiplexed based on the unique barcodes. Paired reads were merged and chimeric sequences were removed using VSEARCH (v. 2.6.1) (40) with the Greengenes reference database (v. 4feb2011) (41) for the 16S library and UCHIME (v. 7.2) (42) reference dataset for the ITS1 library. Chimera-free sequences were clustered into Operational Taxonomic Units (OTUs) at 97% threshold using VSEARCH *de novo*. Both OTU sets were assigned to taxonomy at 97% similarity using the Greengenes database (v. gg_13_8_otus) and Uclust (v. 1.2.22q) (43) in bacterial analysis, and BLAST (44) and UNITE (v. 7.2)^2^ in fungal analysis, resulting in the OTU tables in BIOM format with the singletons discarded.

Further, sparse OTUs with a number of sequences <0.005% of the total sequence number were filtered out of the bacterial dataset (45). PyNAST (v. 1.2.2.) (46) was used to align representative sequences to build a phylogenetic tree using FastTree (v. 2.1.3) (47). QIIME was also used to calculate phylogenetic-based metrics (weighted and unweighted UniFrac distance matrices). Rare taxa with <0.01% relative abundance across all samples were excluded from the fungal OTU table using the Calypso online tool (v. 8.72) (48).

### Statistical Analyses

All analyses were performed using R software (v. 3.5.2) (49) or the Calypso online tool (version 8.72) (48). OTU tables were normalized via total-sum scaling (TSS) followed by centered-log ratio transformation. All data were tested for normal distribution using the Shapiro-Wilk test for normality, and parametric or non-parametric tests were used when appropriate. The presented *p*-values were adjusted for multiple comparison corrections when appropriate. *P*-values below 0.05 were considered statistically significant.

The subjects' clinical characteristics are represented as the mean ± SD, which were determined using the *T*-test. Fisher's exact test was used to assess gender, smoking status, and previous antibiotic use differences.

Alpha-diversity expressed by the Shannon diversity, Richness and the Chao1 indices was calculated and visualized in Calypso. The Shannon diversity index measures the overall diversity (number of present OTUs, evenness), the Richness index expresses the number of present OTUs, and the Chao1 index also measures, besides OTU richness, the ratio of singletons to doubletons to give more weight to rare species (50). The *t*-test and the paired *t*-test were used to determine alpha-diversity differences between study cohorts.

Beta-diversity analyses were performed to explore associations between the microbial composition in the samples and various environmental variables via Analysis of similarities (ANOSIM), Permutational Multivariate Analysis of Variance Using Distance Matrices (ADONIS+) and Redundancy analysis (RDA+) tests were implemented in Calypso. The ANOSIM and ADONIS+ test use selected distance matrices (weighted UniFrac, unweighted UniFrac, Bray-Curtis dissimilarity). ANOSIM is a rank-based test which compares intra-group and inter-group community distances. ADONIS+ is a multivariate test which tests if the variance in microbial composition could be explained by the different explanatory variables (“ALL” and “PAIRS” groups: diagnosis, household, age, BMI, sex, ATB, and smoking status, “Patients” cohort: clinical classifications, treatment type, complication type, immunoglobulin levels). RDA+ is a supervised multivariate method which explores complex associations between microbial composition and different explanatory variables independently on a distance matrix. Selected data associations were visualized via 2D principal coordinate analysis (PCoA) plots using selected distance matrices.

Differences in microbial composition between study cohorts were identified via the: Linear discriminant analysis Effect Size (LEfSe), Differential gene expression analysis based on the negative binomial distribution (DESeq2), and regression analyses, as implemented in Calypso. LEfSe was used to identify taxa potentially associated with health status. LEfSe analysis finds taxa which are most likely to explain the differences between study cohorts. DESeq2, a test developed for count data and small sample cohorts, finds taxa which differ in their relative abundances between cohorts. Correlations between taxa and health status were assessed by regression analysis using the Spearman's correlation coefficient. Taxa which were significantly different between cohorts were visualized via stripchart plots and paired dot plots.

## Results

### Study Population Characteristics

In this study we assigned the participants to two groups. The first main group termed “ALL” was constituted of all 55 participants enrolled in this study, 27 patients with CVID, and 28 healthy controls. The second group (a subgroup within the “ALL” group) termed “PAIRS” was consisted of 16 patients and their 16 partners from the same household, who served as controls to reduce the different environmental impacts on gut microbiota composition. The patient and control cohorts were homogeneous in age, sex, BMI, and smoking status in both groups (Table 1). Previous antibiotic use (more than 1 and <12 months prior to sampling) was significantly higher in the patients' cohort in both the ALL and PAIRS group (Table 1).

### Bacterial Microbiota Differs From Fungal Microbiota

After quality-filtering steps, a total of 20,756,053 *16S rRNA* gene sequence reads and 1,789,802 *ITS1 rRNA* gene sequence reads were obtained from all 55 participants, with an average 377,383 ± 90,742 bacterial and 32,542 ± 10,768 fungal reads per sample. Reads were clustered in 552 and 163 bacterial and fungal OTUs, respectively, at 97% similarity level. Bacterial OTUs were distributed to 86 taxa, of which 63 taxa (68.2% of total reads) were assigned to the genus level. Ten OTUs from ITS1 analysis (0.42% of total reads) were not assigned to the kingdom Fungi, and the remaining 153 fungal OTUs were distributed to 68 taxa at the genus level (92.3% of total reads).

On average, 63 bacterial genera (range: 50–71) and 12 fungal genera (range: 6–21) were detected per sample. The general properties of bacterial and fungal microbiota differed. Fungal microbiota was less rich and more variable than bacterial microbiota. We detected 12 fungal genera singletons, and only two genera (2.9%) were shared by all participants, compared to 25 genera in bacterial microbiota (29.1%) shared across all samples and no singleton genus detected (Table 2). We detected 22 genera and 14 families in bacterial microbiota and 9 genera and 8 families in fungal microbiota with an average abundance over 1%. All taxa above a 1% average relative abundance including numbers of positive samples are listed in Supplementary Table S2.

**Table 2:** Characterization of the bacterial and fungal microbiome properties at genus level.

**Genus Level (*n* = total number)**	**Unique (1 sample)**		**Frequent (>50% samples)**		**Common (all samples)**	
	***n***	**%**	***n***	**%**	***n***	**%**
Bacteria (*n* = 86)	0	0	67	77.9	25	29.1
Fungi (*n* = 68)	12	17.6	9	13.2	2	2.9

*Unique, Number of taxa present in only one sample; Frequent, Number of taxa present in at least half of the samples; Common, Number of taxa present in all samples*.

### Differences in Bacterial but Not Fungal Alpha-Diversity Between CVID and Controls

**Figure 1:**
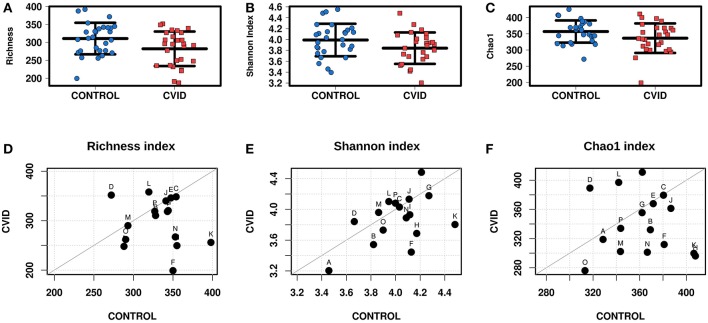
Bacterial alpha-diversity of gut microbiome between CVID patients and healthy controls; Strip chart plots (group “ALL”) and paired dot plots (group “PAIRS”) depict microbiome diversity differences according to the Richness index **(A,D)**, Shannon index **(B,E)**, and Chao1 index **(C,F)**. Each letter in paired dot plots represents one pair of CVID and healthy control from the same household.

The alpha-diversity of the CVID and Control cohorts' gut communities in both groups was evaluated in terms of a number of observed OTUs (Richness), Shannon index and Chao1 index. Generally, the bacterial diversity was ~10 times higher than fungal diversity (Table 3), however, different sequence filtration steps were applied during analyses (see Methods). In bacterial microbiota, all measured alpha-diversity indices were lower in the CVID cohorts than in the Control cohorts, but only the difference in the Richness index was statistically significant in the ALL group (*p* = 0.0256), in contrast to the PAIRS group, where all the differences reached statistical significance (Table 3; Figure 1). In the case of fungal microbiota, alpha-diversity with health status associations were not observed in any group (Table 3).

**Table 3:** Alpha-diversity.

**Diversity index**	**Bacteria**						**Fungi**					
	**All (*n* = 55)**			**Pairs (*n* = 32)**			**All (*n* = 55)**			**Pairs (*n* = 32)**		
	**CVID**	**Controls**	***p*-value^**a**^**	**CVID**	**Controls**	***p*-value^**b**^**	**CVID**	**Controls**	***p*-value^**a**^**	**CVID**	**Controls**	***p*-value^**b**^**
Richness	282.3 ± 48.1	310.8 ± 43.9	**0.0256**	285.2 ± 48.6	319 ± 32.6	**0.0047**	21.3 ± 5.8	20.4 ± 6.6	**0.4531**	22.1 ± 5.9	20.6 ± 7.1	**0.63**
Shannon	3.8 ± 0.3	4 ± 0.3	**0.0621**	3.9 ± 0.3	4 ± 0.3	**0.0097**	1.1 ± 0.5	1.2 ± 0.5	**0.5829**	1.2 ± 0.4	1.1 ± 0.6	**0.56**
Chao1	336.5 ± 45.5	357.0 ± 34.2	**0.0652**	339.6 ± 41.4	361.4 ± 28.4	**0.023**	20.1 ± 6.4	21.4 ± 6.9	**0.4644**	21.1 ± 6.7	21.7 ± 7.1	**0.63**

*Bacterial and fungal alpha-diversity measures characterized by the Richness, Shannon, and Chao1 indices. Mean index values ± standard deviation are shown per cohort (CVID, CONTROLS) in each group (ALL, PAIRS). P-values < 0.05 (bold) are considered significant: (a) t-test; (b) paired t-test*.

### Health Status and Sharing the Same Household Impact the Beta-Diversity

**Figure 2:**
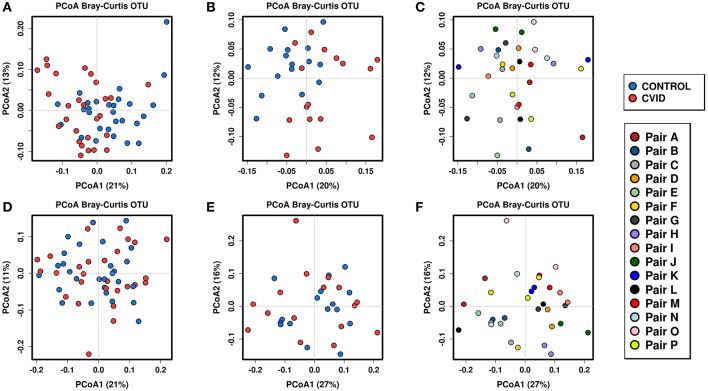
Beta-diversity according to the health status and same household factor; Beta-diversity was calculated from total sum scaling (TSS) normalized OTU data followed by centered log-transformation ratio using the Bray-Curtis distance matrix and visualized using principal coordinate analysis (PCoA) plots. Samples are colored according to the health status (both groups), and the same household (group “PAIRS”). Bacterial beta-diversity: (i) according to the health status: group “ALL” **(A)**, group “PAIRS” **(B)**; (ii) according to the same household (group “PAIRS”) **(C)**. Fungal beta-diversity: (i) according to the health status: group “ALL” **(D)**, group “PAIRS” **(E)**; (ii) according to the same household (group “PAIRS”) **(F)**. Quantitative differences between cohorts are listed in Table 4.

To identify the complex associations between the gut microbiota composition and environmental factors such as health status, same household, age, BMI, sex, antibiotic use, and smoking status, we calculated the samples' beta-diversity using the unweighted and weighted UniFrac distances (only in the bacterial analysis) and the Bray-Curtis dissimilarity distance (bacterial and fungal analyses). The Principal Coordinates Analysis (PCoA) based on Bray-Curtis measures (Figure 2) revealed that the CVID patients' bacterial microbiota was distinct from the healthy controls in both groups (ANOSIM, ALL: *r* = 0.118, *p* = 0.001; PAIRS: *r* = 0.178, *p* = 0.001). These observations were confirmed by multivariate tests ADONIS+ and RDA+, in which the health status was the most significant factor followed by household (PAIRS), and age (ALL) factors (Table 4). Similar results were obtained when using unweighted and weighted UniFrac distance matrices (Supplementary Table S3). Contrary to the bacterial analyses, clustering according to health status was not observed in any fungal analyses (Figure 2). The most significant impact on fungal microbiota composition was the same household factor (ANOSIM, PAIRS: *r* = 0.47, *p* = 0.001), as was also confirmed by ADONIS+ and RDA+ analyses (Table 4). Age and sex were also detected as significant factors; however, their results were inconsistently significant among analyses.

**Table 4:** Bacterial and fungal beta-diversity by environmental variables.

**Parameter**	**ADONIS_+_** **Bray-Curtis**				**Anosim Bray-Curtis**				**RDA_+_**					
	**All (*n* = 55)**		**Pairs (*n* = 32)**		**All (*n* = 55)**		**Pairs (*n* = 32)**		**All (*n* = 55)**			**Pairs (*n* = 32)**		
	***R*^**2**^**	***P***	***R*^**2**^**	***P***	***R***	***P***	***R***	***P***	**Variance**	***F***	***P***	**Variance**	***F***	***P***
**BACTERIA**														
Diagnosis	0.0665	**0.0003**	0.0862	**0.0003**	0.118	**0.001**	0.178	**0.001**	20.50	2.11	**0.001**	30.43	1.86	**0.003**
Household	ND	ND	0.585	**0.0027**	ND	ND	0.352	**0.002**	ND	ND	ND	291.10	1.19	**0.03**
Age category	0.109	**0.006**	0.0887	0.63	0.118	**0.004**	0.156	**0.018**	51.44	1.32	**0.004**	60.46	0.92	0.729
BMI category	0.0608	0.146	0.0402	0.726	0.064	0.077	−0.021	0.609	33.34	1.14	0.149	29.88	0.91	0.716
Sex	0.0133	0.669	0.0221	0.555	−0.008	0.541	0.036	0.139	8.55	0.88	0.772	14.30	0.87	0.742
ATB	0.0166	0.414	0.0159	0.868	0.04	0.234	0.041	0.335	9.23	0.95	0.570	13.99	0.85	0.765
Smoking	0.0396	0.201	0.0421	0.663	0.041	0.333	0.097	0.213	20.61	1.6	0.283	30.3	0.92	0.711
**FUNGI**														
Diagnosis	0.0213	0.29	0.0173	0.528	0.004	0.341	−0.031	0.851	3.10	1.00	0.426	3.73	0.79	0.760
Household	ND	ND	0.701	**0.0007**	ND	ND	0.47	**0.001**	ND	ND	ND	97.05	1.37	**0.033**
Age category	0.102	0.052	0.122	0.0733	0.047	0.096	0.14	**0.04**	15.90	1.29	**0.003**	23.38	1.24	0.220
BMI category	0.0417	0.818	0.0375	0.501	0	0.504	0.032	0.283	11.68	1.26	0.175	7.15	0.76	0.833
Sex	0.0205	0.318	0.0123	0.78	−0.086	0.996	−0.031	0.841	3.43	1.11	0.223	10.11	2.15	**0.021**
ATB	0.0059	0.984	0.0041	0.993	−0.005	0.517	0.042	0.318	2.40	0.78	0.906	2.41	0.51	0.965
Smoking	0.0391	0.415	0.0082	1	−0.08	0.775	−0.097	0.779	5.68	0.92	0.657	4.62	0.49	1

*Beta-diversity was calculated from total-sum scaling (TSS) normalized OTU data followed by centered-log ratio transformation using the Brays-Curtis dissimilarity distance matrix. Analysis of variance using distance matrix (ADONIS+) adjusted for multiple variables: R^2^ value indicates effect size. Analysis of similarities (ANOSIM): R is constrained between the values −1 to 1, where positive numbers suggest more similarity within sites, negative numbers more similarity between sites and values close to zero represent no differences. Bray-Curtis distance independent redundancy analysis (RDA+) adjusted for multiple variables. P-values below 0.05 (bold) were considered significant*.

### CVID Patients Harbor an Altered Bacterial but Not Fungal Gut Microbiota

**Figure 3:**
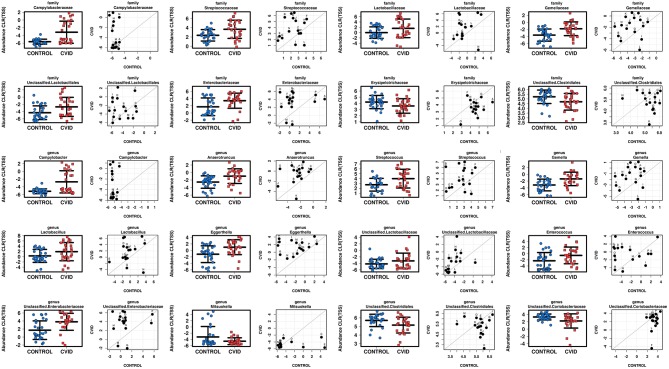
Comparison of total sum scaling (TSS) and centered log-ratio transformation data from bacterial taxa according to health status in group “ALL” (stripchart plots) and group “PAIRS” (paired dot plots). Figure shows 8 and 12 taxa at family and genus level, respectively, significantly altered in CVID patients (Table 5).

To characterize the differences in bacterial and fungal microbiota abundance between CVID patients and healthy controls, we performed three different statistical tests (see Methods): The linear discriminant analysis effect size (LEfSe) (Supplementary Table S4), differential gene expression analysis based on the negative binomial distribution (DESeq2) (Supplementary Table S5), and regression analysis (Supplementary Table S6). Taxa were considered significantly shifted if at least two separate statistical tests discovered these taxa as biomarkers (LEfSe) or significant (*p* < 0.05; DESeq2, regression analysis). This combined analysis showed clear bacterial gut community alterations in CVID characterized by shifts in eight and 12 taxa at family and genus level, respectively (Table 5). These taxa were visualized by stripchart or paired dot plots of both groups (Figure 3). Using the same approach, we detected some shifts in the fungal composition in the context of health status (Supplementary Tables S4–S6); however, the results were not consistent between methods or ceased to be significant after multiple comparison correction adjustments. Only the *Blastobotrys* genus from the *Trichomonascaceae* family remained significantly associated with CVID, although it was present in only seven samples.

**Table 5:** Bacterial taxa at genus and family level significantly associated with health status across at least two of three statistical tests.

**Selected taxa**	**Associated group**	**LEfSe LDA score**	**DESeq2 p-value (FDR)**	**Regression analysis *p*-value**
**FAMILY LEVEL**				
Campylobacteraceae	CVID	3.09	0.0000000013	0.0013
Streptococcaceae	CVID	3.96	0.0000021	0.0074
Lactobacillaceae	CVID	NS	0.00011	0.05
Gemellaceae	CVID	2.95	0.00014	0.00078
Unclassified.Lactobacillales	CVID	NS	0.013	0.0097
Enterobacteriaceae	CVID	3.68	NS	0.0089
Erysipelotrichaceae	CONTROL	3.73	0.019	0.031
Unclassified_Clostridiales	CONTROL	4.12	0.042	0.01
**GENUS LEVEL**				
Campylobacter	CVID	2.27	0.0000000063	0.0012
Anaerotruncus	CVID	2.44	0.0000044	0.00086
Streptococcus	CVID	3.95	0.0000063	0.012
Gemella	CVID	2.29	0.00052	0.00096
Lactobacillus	CVID	NS	0.00073	0.038
Eggerthella	CVID	2.61	0.0047	0.0013
Unclassified.Lactobacillales	CVID	NS	0.0047	0.0092
Enterococcus	CVID	NS	0.0047	0.02
Unclassified_Enterobacteriaceae	CVID	3.72	NS	0.0015
Mitsuokella	CONTROL	3.02	0.00014	NS
Unclassified_Clostridiales	CONTROL	4.11	0.0039	0.0092
Unclassified_Coriobacteriaceae	CONTROL	3.22	0.026	0.007

*Taxa are listed by the DESeq2 significance in each cohort. LEfSe, The linear discriminant analysis (LDA) effect size; DESeq2, Differential gene expression analysis based on the negative binomial distribution; NS, Not significant*.

### More Severe CVID Phenotype and Unmeasurable Serum IgA Levels Are Associated With More Reduced Bacterial Alpha-Diversity

The CVID patient cohort (*n* = 27) was further analyzed for phenotype severity, serum immunoglobulin (IgA, IgM) levels, complication occurrence and its association with gut microbiota diversity (Supplementary Table S1). Patients were characterized in this study using disease classifications according to Ameratunga (1) and Chapel (34) (Supplementary Table S1). The CVID phenotype severity in the Ameratunga category was relatively assessed in our CVID cohort using the median as a threshold to obtain two groups—less severe (*n* = 15) and more severe (*n* = 12) phenotype. The CVID phenotypes in the Chapel category were divided into two main groups “Infection only” (*n* = 15) and “Complications” (*n* = 12) according to previously defined criteria (34).

**Figure 4:**
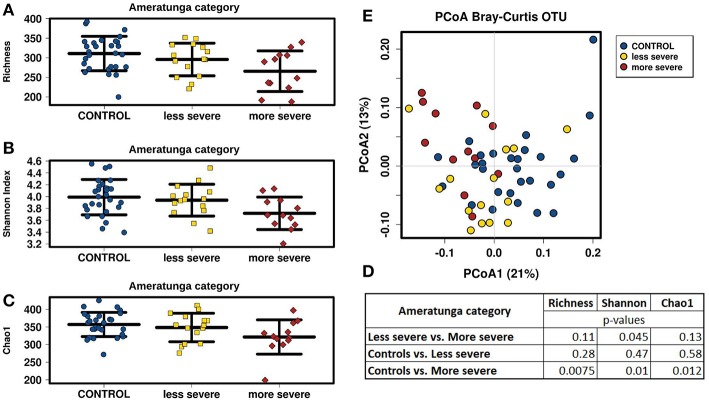
CVID phenotype severity comparison; CVID patients with less severe (*n* = 15) and more severe (*n* = 12) disease phenotype assessed by “Ameratunga category” (Supplementary Table S1) were compared to Control cohort. Stripchart plots depict bacterial microbiome diversity differences according to the Richness index **(A)**, Shannon index **(B)**, and Chao1 index **(C)**. *P*-values were calculated using t-test **(D)**. Principal coordinate analysis (PCoA) plot of beta-diversity depict clustering according to the health status and CVID phenotype severity **(E)**.

We divided CVID cohort into two groups according to the CVID phenotype severity in the Ameratunga category and compared them with the Control cohort (Figure 4). We observed that less severe CVID phenotype was comparable to the controls in alpha-diversity measurements, although alpha-diversity tended to be slightly lower in CVID. Contrary to the less severe phenotype, all alpha-diversity indices in the more severe phenotype were significantly decreased than in the Control cohort (Figures 4A–D). CVID phenotype differences were also reflected in beta-diversity analysis (Figure 4E). The less severe phenotype group overlapped the Control cohort more, indicating similar microbiota composition, and the more severe phenotype tended to cluster separately, suggesting greater differences in microbiota composition. We also observed significant differences in alpha-diversity between the controls and the “Complications” subgroup assessed by Chapel category (Supplementary Table S7). Differences in fungal alpha-diversity associated with the mentioned classifications were not detected.

Next, bacterial alpha-diversity was lower in patients with unmeasurable serum IgA levels (<0.07 g/l) (Supplementary Figure S1), but only the Chao1 index indicates a significant decrease (*p* = 0.015). However, only four patients had measurable serum IgA levels in a sampling day.

We did not observe any other conclusive differences in bacterial or fungal alpha-diversity in a complication occurrence context (bronchiectasis, autoimmunity, splenomegaly, chronic diarrhea, atrophic gastritis, and nodular hyperplasia) (Supplementary Table S8), treatment administration (antibiotic use, substitution therapy type or immunosuppression), or serum IgM levels, however, data analyses in most groups/categories may be burdened by a small number error. Therefore, interpreting the results of these analyses is difficult and more samples would be needed to analyze these complications to resolve whether gut microbiota is affected or not.

## Discussion

In this study, we attempted to: (i) Assess whether the bacterial and/or fungal gut microbiota is affected in CVID by comparing the gut microbiota composition between CVID patients and healthy control cohorts in two groups constituting all participants and case-control pairs who shared the same household, respectively; and (ii) Expand general knowledge about fungal gut microbiota composition, since gut fungi are still relatively understudied and new findings are needed regardless of the study's primary purpose. To achieve these goals, we used alpha- and beta- diversity measurements alongside taxonomic comparisons. Alpha-diversity shows how many and how many different microbes can be found within one sample. Beta-diversity shows how these samples or groups of samples (i.e., CVID vs. Controls) vary against each other, and taxonomic comparisons specifically show these differences.

Our results indicate that CVID patients harbor less diverse and significantly altered bacterial communities in their gut. It also appears that the gut bacterial microbiota is associated with the CVID phenotype severity, which is in concordance with the previous study's outcome that bacterial microbiota may be involved, at least partially, in systemic immune activation in CVID (26). Contrary to bacterial analyses, only one relatively underrepresented fungal genus *Blastobotrys* was associated with CVID in our study, however, this genus was present in only a few samples, and thus mycobiota statistical analyses exhibited no conclusive health status associations indicating that fungi are probably not relevant contributors to the CVID phenotype. In parallel, we examined a group of 16 case-control couples who shared the same household to decrease the different environmental impact on gut microbiota composition. We found that sharing the same household is a strong factor influencing the microbiome data. While in the case of bacterial analyses, the household's impact was strong but did not outweigh the influence of the health status on microbiota diversity, in the case of fungal analyses, the household was the most significant diversity determining factor.

### Gut Microbiota in CVID

In last few decades we have expanded our understanding of the role of the human gut microbiome in health and disease. Many studies associated gut microbiomes not only with gastrointestinal tract diseases, but also with extra-intestinal conditions including immune system disorders [reviewed in (51)]. Nevertheless, studies linking the gut microbiome to CVID are still rare. To the best of our knowledge, only three studies have focused on gut microbiota composition in CVID patients (25, 26, 28), and none of them analyzed gut mycobiota or used paired controls from others sharing the same household. We provide a new view on CVID bacterial microbiota alongside previous findings' comparisons, and at the same time provide unique findings from CVID gut mycobiota analysis. Our results from bacterial analyses could be most easily compared to Jørgensen et al. (26) study results since other two mentioned studies (i) used different methodology and/or sample type, (ii) analyzed a small number of CVID patients, and (iii) did not report the full list of CVID taxa abundance for comparison.

#### Bacterial Gut Microbiota in CVID

In agreement with previous findings (25, 26), we observed reduced bacterial diversity in CVID patients. Recently reduced bacterial alpha-diversity was recognized as a possible universal gut microbial biomarker of common human intestinal diseases (52). The main idea of the hypothesis that higher microbiota diversity is connected with better health status lies in the greater ability to adapt to possible perturbations. However, diversity alone may be a poor marker of disease (53), and sometimes “the higher microbiota diversity the better” hypothesis does not hold true (54). On the other hand, low diversity combined with microbial dysbiosis might have a biological significance (53). We detected perturbations in taxa abundance in CVID patients, also reflected the in the beta-diversity analyses, where the CVID cohort tended to cluster separately from the Control cohort. For example, increased *Streptococcaceae, Lactobacillaceae*, and *Enterobacteriaceae* families in our CVID cohort alongside reduced diversity have been linked to other diseases inside (52), and also outside (55) the gut, suggesting the existence of the common dysbiosis feature (56). It is also noteworthy to mention that human-pathogenic genus *Campylobacter* was exclusively present in our seven CVID patients with no GIT symptoms reported suggesting their different susceptibility to *Campylobacter* colonization and the inability of their immune system to adequately respond to it (57).

We also suggest that reduced diversity in CVID patients in our dataset has biological significance, since patients with a more severe CVID phenotype displayed more decreased alpha-diversity than patients with a less severe CVID phenotype. CVID patients also clustered more distantly in beta-diversity analyses, which indicates different microbiota composition. Next, alpha-diversity was also lower in patients with unmeasurable serum IgA levels; however, as low IgA levels are typical for CVID, there were only four with measurable serum IgA for comparison in our group. These results partially correspond with results of Jørgensen et al. (26), where CVID patients with complications, and/or with decreased plasma IgA levels also had reduced alpha-diversity when compared with patients suffering from infections only, and/or with normal plasma IgA levels. However, plasma/serum IgA levels do not necessarily correspond with secretory IgA levels, which are known to impact CVID gut microbiota directly (28). In their study, Shulzhenko et al. observed that CVID patients with low levels of secretory IgA developed CVID enteropathy whereas patients with normal secretory IgA levels did not. Despite the secretory IgA antibodies being polyreactive, they are only able to coat a restricted spectrum of microbes (58). Species known to be IgA-coated mainly include members of the *Proteobacteria* phylum (i.e., *Enterobacteriaceae*) and *Firmicutes* phylum (i.e., *Lactobacilli*) (19). These members are also relatively elevated in our CVID cohort, and it can be speculated that the “CVID dysbiosis” may be associated with depleted secretory IgA. Nevertheless, these results should be verified in larger patient cohorts with known plasma/serum and secretory IgA levels. Furthermore, it has been very recently described that systemic IgG and secretory IgA bind a common spectrum of gut microbiota, therefore secretory IgA depletion alone may often remain asymptomatic since IgG may provide a second level of protection (59). However, IgG protection seems to be personalized, and although immunoglobulin replacement therapy administered to CVID patients contains an extended set of anti-commensal IgG, it seems to bind CVID microbiota less efficiently, and thus may lead to microbiota dysregulation and possible dysbiosis (59).

We further compared all taxa significantly altered in our study to the CVID microbiota results from other studies (25, 26, 28). Our main results were mostly in agreement with these studies; however, some contradictories were detected. First, Shulzhenko et al. (28) did not observe any significant differences in bacterial abundance between CVID and controls, which is in contrast to our results; however, a low number of duodenal biopsies had been evaluated in their study so the results are not easily comparable. Second, Fadlallah et al. (25) described low *Actinobacteria* phylum diversity in their CVID patients with very low secretory IgM levels, which was also not observed in our study, however, we did not measure intestinal immunoglobulin levels; therefore we cannot exclude CVID phenotype differences' impact in our cohorts. Last, Jørgensen et al. (26) calculated a “CVID specific dysbiosis index” using several taxa significantly altered in their CVID cohort. These taxa did not differ from the healthy control group in our study except taxa from *Bacilli* class and *Enterobacteriaceae* family. Furthermore, we even detected *Anaerotruncus* and *Eggerthella* genera to be significantly increased in CVID, which was opposite to their results. Therefore, we suppose that the taxa differences may not be strongly associated with the CVID phenotype, or other confounding factors may have influenced the resulting outcome such as (i) geographical origin, (ii) different CVID spectrum analyzed, and (iii) other factors including methods and cohort size differences.

In parallel, within our participant group, we analyzed 16 CVID patient-partner pairs sharing the same household to diminish the impact of different environmental factors. We confirmed that most pairs were more similar to each other than to the strangers in terms of bacterial microbiota composition, which corresponds to previous findings (60, 61). However, despite the similarities between the pairs, the health status remained the most significant feature in beta-diversity analyses. Furthermore, health status differences become even more visible in alpha-diversity analyses as well as in taxa abundance analyses when performing paired tests (see Methods). These results indicate that despite the high inter-individual and inter-pair variability, the CVID phenotype was well reflected in the bacterial data.

#### Fungal Gut Microbiota Properties

Fungal gut microbiota research lags way behind those for bacteria, although it has recently attracted more attention. One of the Human Gut Microbiome Project (HMP) studies has widened our knowledge about “healthy” fungal gut microbiota (62), and there are many other studies correlating gut mycobiota composition and its role in various health issues and disease conditions [reviewed in (63)]. There, gut fungi were characterized as low in diversity and very variable between individuals (62, 64, 65). In line with previous findings, we observed a higher fungal variability between samples and ~10 times lower fungal diversity than the bacterial analyses in our study. On average, one participant harbored 12 fungal genera; however, only a few fungal genera were shared by more than a third of participants, and these genera represented a core mycobiome in this study. The most abundant core mycobiome genera were identified as *Saccharomyces, Penicillium, Dipodascus, Debaryomyces, Candida, Pichia, Aspergillus, Rhodotorula*, and *Hanseniaspora*. The mentioned fungal genera were also found in various abundance in the stool samples by others (62, 64, 66), however, some genera such as *Malassezia* or *Cryptococcus*, commonly detected elsewhere (62, 67), were not present in our samples. Inconsistencies in results may be partially explained by the different methodology, such as different fungal primers used across the studies. For example, primer pair targeting the ITS1 region, which was used in this study, is known to underrepresent *Malassezia* genus abundance (68). The current nomenclatural changes implemented in the UNITE database (69, 70) provide another explanation and affect many fungal taxa including *Cryptococcus* genus, which was taxonomically assigned to its homotypic synonyms (*Filobasidium, Vishniacozyma, Naganishia, Filobasidium*, and *Cutaneotrichosporon*) in our data. Moreover, geographical-based differences might also impact the results similarly as described in bacterial gut microbiota research (71).

#### Fungal Gut Microbiota in CVID

To the best of our knowledge, this is the first study analyzing gut mycobiota in CVID patients; therefore, there is no other similar dataset for an appropriate comparison. First, we report no differences in fungal alpha-diversity between patients and controls; however, the impact of fungal alpha-diversity on human health is still unclear. Overall fungal alpha-diversity was ~10 times lower than bacterial alpha-diversity, and contradictories in the same disease-related alpha-diversity measurements were reported (29, 72), suggesting that fungal alpha-diversity may not be critical in disease evaluation. Second, we did not observe any obvious taxonomic differences between patients and controls in our study, indicating that gut mycobiota may not affect the CVID phenotype, at least not in a detectable way. Nevertheless, we observed one fungal CVID phenotype association, specifically *Blastobotrys* genus, although it was present in only six CVID and one control sample. This genus was not previously reported in the context of the human gut, and therefore it may not be a true gut colonizer. On the other hand, *Blastobotrys* species are capable of growing in 37°C, and they were reported to cause invasive fungal infections, although extremely rare, in immunocompromised patients (73). Therefore, this possible association deserves deeper examination. All in all, we did not find any convincing associations between gut mycobiota and CVID phenotype, however, since the properties of fungal composition differ from bacterial composition tremendously, as described above, we cannot exclude the fungal impact may lie in other aspects than in taxa diversity or abundance differences. For example, various fungal species overgrowth, which was not measured in our study since only taxa relative abundance was assessed, has been often found to correlate with disease state in other similar mycobiota studies (29), and thus our study may not have revealed all possible associations.

Similar to bacterial analyses, we further evaluated a smaller “PAIRS” group in terms of mycobiota diversity, and we did not observe any health status associations in this dataset, which corresponds with the results stated above. Instead, most of the paired samples clustered close to each other in beta-diversity analyses, indicating a similar fungal mycobiota composition in members of the same household. It agrees with the correlative associations observed both in a mice study, where mice from the same cage were colonized with specific fungi differing from other cages (74), and in only one human study, focusing on human mycobiota transfer from mother to offspring (75). Other data from human research are unfortunately lacking, therefore the biological relevance of these correlations remains unknown. Thus, future studies using paired samples from the same household could provide a new research opportunity to assess whether gut mycobiota is truly capable of colonizing human gut and can be transferable between partners, or is only the transiently present fungal DNA originating from the environment, as was previously outlined elsewhere (76).

## Conclusion

In summary, our study extends previous findings of the correlation between the bacterial gut microbiota and CVID and provides new insights into overall gut mycobiota composition. Furthermore, we reveal the strong impact of sharing the same household on bacterial and fungal microbiome data, although weaker than that of CVID diagnosis in bacterial assessment. This suggests that paired samples serving as controls in future studies would provide a better resolution between disease-related dysbiosis and other environmental confounders. The cause of CVID still remains unknown in most cases; however, gut microbiota may be one of the missing environmental drivers contributing to some of the symptoms and their severity. Although, due to the high CVID heterogeneity and the limits of current methodology, it is still unclear whether CVID results in dysbiosis and/or dysbiosis contributes to the CVID phenotype. Therefore, the larger metacentric studies including gut microbiota profiles, metagenomics, metabolomics, as well as more detailed and topical immunological evaluation will be needed to further establish the relevance of gut microbiota in CVID.

## Data Availability

The raw sequences of datasets for this study can be found in the European Nucleotide Archive repository, http://www.ebi.ac.uk/ena under accession number: PRJEB32265.

## Author Contributions

TF, DS, JL, KF, and JB conceived the initial project design and discussed project progress. IK provided partial assistance with experimental project workflow. KF, MC, and EN processed stool samples. KF performed all experiments and statistical analyses of the data with significant contributions from EN and HG. MR performed the bioinformatics analysis of the data. ML provided bioinformatics assistance. PK and JL provided stool samples alongside patients' clinical and laboratory characteristics. KF, HG, and TF wrote the manuscript and EN, JB, DS, IK, and JL significantly contributed to the final preparation of the manuscript. All authors revised and approved the final manuscript.

### Conflict of Interest Statement

The authors declare that the research was conducted in the absence of any commercial or financial relationships that could be construed as a potential conflict of interest.

## Footnotes

^1^http://www.earthmicrobiome.org/protocols-and-standards/its/

^2^PlutoF biodiversity platform Available at: https://plutof.ut.ee/#/datacite/10.15156%2FBIO%2F587476
