# Supplementary material for: Bacterial but Not Fungal Gut Microbiota Alterations Are Associated With Common Variable Immunodeficiency (CVID) Phenotype
Source: Front Immunol. 2019 Aug 13;10:1914. doi: 10.3389/fimmu.2019.01914 (PMC6700332; doi:10.3389/fimmu.2019.01914)
Supplement: Supplementary file 1 [file Data_Sheet_1.docx]

Supplementary Material


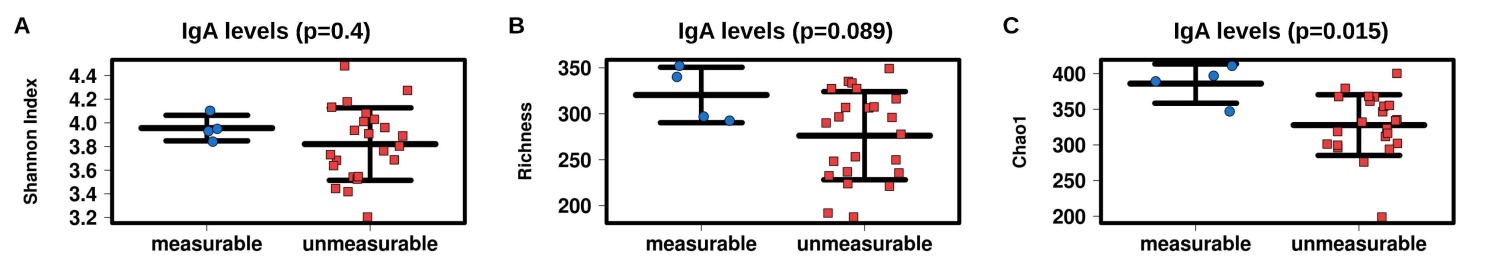


**Supplementary Figure S1.** Bacterial alpha-diversity of gut microbiome between CVID patients with measurable serum IgA levels (≥ 0.07 g/l) and unmeasurable serum IgA levels (< 0.07 g/l). Stripchart plots depict microbiome diversity differences according to the Shannon index (A), Richness index (B), and Chao1 index (C). P-values were calculated using t-test.
